# Pseudo-Pemphigoid Gestationis Eruption Following SARS-CoV-2 Vaccination with mRNA Vaccine

**DOI:** 10.3390/dermatopathology9030025

**Published:** 2022-06-24

**Authors:** Caroline de Lorenzi, Gürkan Kaya, Laurence Toutous Trellu

**Affiliations:** 1 Department of Dermatology and Venereology, Geneva University Hospitals, Rue Gabrielle-Perret-Gentil 4, CH-1205 Geneva, Switzerland; gkaya@hcuge.ch (G.K.); laurence.trellu@hcuge.ch (L.T.T.); 2 Department of Clinical Pathology, Geneva University Hospitals, CH-1205 Geneva, Switzerland

**Keywords:** pemphigoid gestationis, bullous auto-immune disease, mRNA vaccine, SARS-CoV-2

## Abstract

Auto-immune reactions, including auto-immune bullous disease, have been reported following SARS-CoV-2 virus vaccination. Few cases of bullous pemphigoid are described, but there has been no case of pemphigoid gestationis. We report the first case here.

## 1. Case Presentation

**Figure 1:**
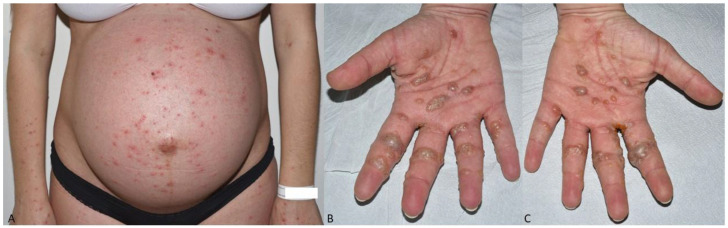
(**A**) Exanthema with erythematous macules and papules on trunk and limbs, with few pustules. (**B**) and (**C**) Tense bullae on palms.

A 38-year-old primigravida woman at 38 weeks’ gestation, without any previous medical history, was admitted to our dermatology unit after presenting pruriginous disabling skin lesions for 3 weeks. They initially appeared on the palms and the soles, and then rapidly spread across the trunk. She did not report any allergy, atopy, new medication, or previous infectious episode, but received the second dose of the SARS-CoV-2 mRNA Pfizer vaccination 3 days before. She presented an exanthema with erythematous macules and papules on trunk and limbs, with few pustules, as well as tense bullae on her palms and soles (Figure 1A–C).

**Figure 2:**
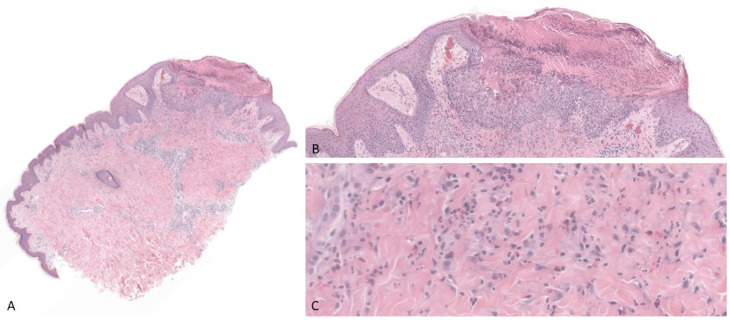
(**A**,**B**) Histology showing an erosion covered by a crust containing neutrophils and a dermal inflammatory infiltrate (H&E, original magnification: 1.6× and 6.5×, respectively). (**C**) Inflammatory infiltrate with neutrophils and numerous eosinophils (H&E, original magnification: 26×).

Lab tests revealed the presence of leukocytosis (13.1 G/L) with eosinophils (0.55 G/L), and liver tests, biliary acids, and total IgE were in the range. Bacterial smear, herpes simplex, zoster virus PCR, and enterovirus PCR were negative, and we excluded fungi on direct examination and culture of a roof bullae. A skin biopsy of a flank lesion showed an erosion covered by a crust containing neutrophils and a dermal inflammatory infiltrate with neutrophils and numerous eosinophils (Figure 2A–C). PAS, Gram, and Grocott stainings were negative. Direct immunofluorescence, taken on a lesion localized on the abdomen, revealed C3 on a few vessels with granular deposits. Indirect immunofluorescence and ELISA for anti-BP180 and -BP230 were both negative.

Due to her chronology of polymorphous cutaneous lesions, after clinicopathological correlation, we confirmed a bullous pseudo-pemphigoid gestationis reaction following mRNA Pfizer vaccination. Differential diagnosis included a pemphigoid gestationis, a pruritic urticarial papules and plaques of pregnancy, and dyshidrotic eczema. Clinical evolution of the mother was rapidly good under topical corticosteroids (clobetasol and then mometasone creams) and oral desloratadine. A simultaneous obstetrical follow-up did not reveal any anomalies and she was transferred to the obstetric unit 8 days later where she gave birth to a healthy baby.

## 2. Discussion

Our patient presented a bullous skin reaction clinically similar to a pemphigoid gestationis (PG) which occurred 3 days after the mRNA Pfizer vaccination. We excluded an auto-immune blistering disease (AIBD) and retained the diagnosis of pseudo-PG eruption induced by the second dose of the mRNA COVID-19 vaccine (Pfizer).

Numerous skin reactions have been described following mRNA COVID-19 vaccination with a broad spectrum reported from local injection site to unusual cutaneous reactions [1,2]. Bullous reactions following SARS-CoV-2 vaccination have also been reported. They include non-immune bullous reactions and bullous drug reactions [3], but AIBD triggered by anti-SARS-CoV-2 vaccines have also been published. Among them, few cases of bullous pemphigoid (PB) are described in the literature with mRNA Moderna and Pfizer vaccines, as well as the adenovirus-based AstraZeneca vaccine [4,5]. It was hypothesized that autoimmune reaction could be elicited by molecular mimicry between human extracellular heat shock proteins and immunologic viral proteins; however, using serological analyses on different patients, recent reports have demonstrated that anti-SARS-CoV-2 antibodies do not react with pemphigus or pemphigoid auto-antigens [6]. Moreover, post-vaccination BP has also been reported following other vaccinations (e.g., influenza, among others), potentially representing a non-specific immune-mediated activation [2].

Concerning the latency between the onset of cutaneous symptoms and SARS-CoV-2 vaccine, among 130 cases described by McMahon et al. who have received two vaccine doses, only 22% reported a reaction to the first dose and 58% to the second dose, with a median delay time of 7 days after the first dose and a shorter time after the second one, but the occurrence of generalized eruption was also reported 1 to 2 weeks after the second dose [1,2]. Autoimmune bullous reactions, such as BP, also seem to appear later after injection, probably reflecting the period of antibodies production [2].

Histopathology can help to improve the diagnosis with different patterns described depending on the skin reaction, with epidermal spongiosis, interface changes, mixed-cell infiltrates, and eosinophils being common findings [2]. In the case of suspected AIBD, direct immunofluorescence studies should be performed to distinguish between non-immune and immune bullous reactions [2], as well as indirect immunofluorescence and research on circulating autoantibodies.

Managing skin reactions following SARS-CoV-2 vaccination is symptomatic and includes antihistamines, topical corticosteroid, and pain relief, with systemic cortiotherapy for severe cases or extensive AIBD-induced cases [1,7]. The second vaccine dose is contraindicated in patients presenting an immediate hypersensitivity reaction (urticarial, flushing, and angioedema) [1]. However, the SARS-CoV-2 vaccination of patients with AIBD is encouraged [6], although we must keep in mind that we cannot predict if someone genetically predisposed will develop or aggravate autoimmunity after SARS-CoV-2 infection or immunization [8].

The different diagnosis of bullous eruption in a pregnant woman includes PG. It is a rare AIBD, associated with HLA DR3 and DR4 alleles, and occurs during the 2nd–3rd trimester [9]. PG is clinically similar to BP and is caused by IgG autoantibodies against BP180 epitopes, but patients with predominantly acral vesicles seem to not present NC16A BP180 antibodies [9,10]. It can be complicated with spontaneous abortions, stillbirth, preterm delivery, and small gestational age, and neonatal PG is seen in 2.8–15% of newborns due to placental transfer of autoantibodies [9]. First-line PG treatment includes antihistaminic therapy, topical corticosteroids, and, in severe cases, oral prednisolone [9]. It spontaneously resolves after pregnancy, but recurrences can be seen in subsequent pregnancies [9]. PG following SARS-CoV-2 vaccination has never been reported before.

In conclusion, the causality between the auto-immune bullous reaction and vaccination against SARS-CoV-2 is still unclear, and there is still room for further progress in the future. Dermatopathological clues can improve diagnosis, as well as usual immunofluorescence studies and blood tests used for circulating autoantibodies. Given the rarity of these events, a first vaccination of SARS-CoV-2, as well as boosters, should be encouraged in patients with AIBD while remaining vigilant.
